# Two-step discriminant analysis based multi-view polarimetric SAR image classification with high confidence

**DOI:** 10.1038/s41598-022-09871-w

**Published:** 2022-04-08

**Authors:** Maryam Imani

**Affiliations:** Faculty of Electrical and Computer Engineering, Tarbiat Modares University, Tehran, Iran

**Keywords:** Electrical and electronic engineering, Environmental sciences

## Abstract

Polarimetric synthetic aperture radar (PolSAR) image classification is a hot topic in remote sensing field. Although recently many deep learning methods such as convolutional based networks have provided great success in PolSAR image classification, but they need a high volume of labeled samples, which are not usually available in practice, or they cause a high computational burden for implementation. In this work, instead of spending cost for network training, the inherent nature of PolSAR image is used for generation of convolutional kernels for extraction of deep and robust features. Moreover, extraction of diverse scattering characteristics contained in the coherency matrix of PolSAR and fusion of their output classification results with a high confidence have high impact in providing a reliable classification map. The introduced method called discriminative features based high confidence classification (DFC) utilizes several approaches to deal with difficulties of PolSAR image classification. It uses a multi-view analysis to generate diverse classification maps with different information. It extracts deep polarimetric-spatial features, consistent and robust with respect to the original PolSAR data, by applying several pre-determined convolutional filters selected from the important regions of image. Convolutional kernels are fixed without requirement to be learned. The important regions are determined with selecting the key points of image. In addition, a two-step discriminant analysis method is proposed to reduce dimensionality and result in a feature space with minimum overlapping and maximum class separability. Eventually, a high confidence decision fusion is implemented to find the final classification map. Impact of multi-view analysis, selection of important regions as fixed convolutional kernels, two-step discriminant analysis and high confidence decision fusion are individually assessed on three real PolSAR images in different sizes of training sets. For example, the proposed method achieves 96.40% and 98.72% overall classification accuracy by using 10 and 100 training samples per class, respectively in L-band Flevoland image acquired by AIRSAR. Generally, the experiments show high efficiency of DFC compared to several state-of-the-art methods especially for small sample size situations.

## Introduction

Polarimetric synthetic aperture radar (PolSAR) as a high resolution and multi-channel imaging equipment allows a wide range of remote sensing applications especially for target detection and land cover classification^1,2^. In some works, SAR data is used beside the optical multispectral image to provide an improvement in land cover classification^3^. Due to crosstalk among polarization channels, phase bias and channel imbalance, there are polarimetric distortions in a PolSAR system. The researchers in^4^ tried to estimate and minimize the mentioned distortions through polarimetric calibration of PolSAR images. There is rich scattering information in a PolSAR image. Many target decomposition methods have been introduced to extract the polarimetric and scattering characteristics of PolSAR data^5,6^. One of the main challenges of this subject is scattering ambiguity. For example, the cross-polarized scattering can be caused by both vegetation and rotated dihedrals in urban areas. To deal with this difficulty, the extended models have been introduced in^7,8^ for four-component decomposition of PolSAR images with considering the cross scattering of corner reflectors in oriented buildings.

Beside the polarimetric and scattering features, which can be extracted by various target decomposition methods, the textural features have important role for an accurate PolSAR image classification. Polarimetric parameters beside the grey level co-occurrence matrix features are used for classification of river ice by applying a random forest classifier on dual PolSAR data acquired by Sentinel 1 in^9^.

Color features extracted from pseudo-color images obtained by color coding methods have been used beside the polarimetric and texture features for PolSAR image classification in^10^. The results have shown significant role of the color features in improvement of classification accuracy. High dimensional decomposition features are fused with texture features through the composite kernel and hybrid random field model (CK-HDRF) in^11^. Different PolSAR feature spaces such as polarimetric data scattering and target decomposition spaces are fused to achieve a distinctive feature set for PolSAR image classification in^12^. The suggested method in^12^, called modified tensor distance based multi-view spectral embedding (MTD-MSE) uses a tensor based multi-view embedding for feature fusion. Complementary characteristics of different views are exploited for extraction of an appropriate low dimensional feature set. An online deep forest model has been proposed in^13^ for considering different sources or feature spaces regarded as multi-view. The experiments have shown classification improvement due to multi-view, multi-feature or multi-frequency PolSAR images, and also due to fusion of PolSAR data with optical images.

In addition to improvement of appropriate features in a PolSAR data classification, it is a serious affair to suitably deal with the main challenges of PolSAR data classification. Speckle noise and limited available training samples are among the main challenges of PolSAR image classification. Including spatial information and utilizing super-pixel and object based methods have been proposed to deal with the first difficulty^14–16^. The use of semi-supervised approaches and active learning are among the suggested methods to deal with the second difficulty^17–19^. An online active extreme learning machine (OA-ELM) has been proposed in^17^, which its aim is to improve training efficiency, classification accuracy and also the generalization ability. Support vector machine (SVM) can be an appropriate classifier when limited training samples are available^20^. The pairwise proximity function SVM is proposed in^21^ for time series analysis of dual polarization SAR for crop classification.

Much recently published works have used deep learning for PolSAR image classification^22–25^. Deep convolutional neural networks (CNNs) have been suggested for PolSAR image classification in^26^. An improved version of CNN has been proposed in^27^, which utilizes the complex information of PolSAR and also the abilities of 3D-CNN for simultaneous extraction of polarimetric and spatial features. Complex valued 3D-CNN called as CV-3D-CNN has shown superior classification results compared to previous CNN based methods. Efficiency of CNN is tested on multi-resolution dual-Pol images for land cover classification in^28^. A new structure of CNN is proposed for PolSAR image classification in^29^. The low frequency components of PolSAR are used for noise reduction. In addition, the fuzzy clustering maps of the scattering characteristics of PolSAR are concatenated to the texture features to result in a rich spatial-physical feature cube of the PolSAR image.

A PolSAR image classification method is proposed in this work, which utilizes several approaches to deal with difficulties of PolSAR data classification. The proposed method, called discriminative features based high confidence classification (DFC), considers diagonal, real and imaginary parts of the coherency matrix as three views of PolSAR data containing different information. DFC includes diverse scattering features for PolSAR data classification by applying a multi-view analysis. Each view is individually analyzed. Two pre-determined convolutional layers are used for polarimetric-spatial feature extraction in each view. In contrast to convolutional filters in CNNs, which contain hyper-parameters and need training samples to learn, the pre-determined convolutional filters, selected among the important regions of the original image, are used in DFC. So, the use of them not only is simple and fast but also results in consistent feature maps accordance with the PolSAR image. The previous work^30^ suggested the use of fixed convolutional kernels. But it selects kernels randomly from the entire scene. In this work, the convolutional kernels are selected among the important regions of the image, which lead to extraction of valuable features. To remove redundant features and utilize features with maximum differences with respect to each other and also to maximize class discrimination, a two-step discriminant analysis method is proposed for feature reduction. The reduced feature maps of each view are used for generation of a classification map in that view. Finally the classification maps are fused reliably by involving the neighborhood information. The result of this decision fusion is an accurate classification map. Some contributions of this work are represented in the following:

1. Three views with multiple scattering characteristics are used for PolSAR image analysis to achieve diverse classification maps.
2. The convolutional kernels are selected among the important regions of the PolSAR image. Applying these convolutional kernels can extract important spatial features consistent with contents of the assessed scene without spending high computational burden or using any training set.
3. A reduced feature space with minimum redundancy and maximum class-separability is provided.
4. Associated with each classification map generated from each view of the PolSAR image, a confidence map is computed, which leads to an accurate decision fusion.

The proposed method is introduced in Sect. 2. The experimental results are discussed in Sect. 3 and finally Sect. 4 concludes the paper.

## Proposed classification method

The proposed method called discriminative features based high confidence classification (DFC) uses four contrivances to increase the classification accuracy of the PolSAR data: 1. multi-view (MW) analysis, 2. selection of important regions (IRs) for spatial filtering, 3. two-step discriminant analysis (DA) and 4. high confidence (HC) decision fusion.

The coherency matrix of each pixel of PolSAR image contains diagonal, real and imaginary parts, which each of these parts has obviously different characteristics and diverse features. So, each part is considered as a view of PolSAR data that is individually analyzed. To extract deeper and more robust features, several fixed and pre-determined kernels are convolved to each view of the PolSAR data. The convolutional kernels are selected from the most important regions of the PolSAR image. Difference of Gaussian function^31^ is used to determine the important regions. The extracted feature maps have high dimensionality which may contain redundant information. To extract the least overlapping and the most discriminative features, a two-step discriminant analysis based feature reduction method is proposed. The extracted features of each view generate a classification map by utilizing SVM as a classifier. Associated with each classification map, a confidence map is also generated, which is used for decision fusion for providing the final classification map. The proposed DFC method has the following advantages:

1. DFC is efficient using limited training samples because it uses the fixed and pre-determined convolutional kernels without requirement to be learned. In addition, a two-step discriminant analysis method for dimensionality reduction is applied to the polarimetric-spatial cube, which is nanparametric with low sensitivity to the training set size.
2. Applying pre-determined convolutional filters, selected from the most important regions of the PolSAR image, not only degrades the speckle noise but also extracts robust spatial features consistent with the PolSAR image.
3. DFC utilizes the discriminative, informative and rich polarimetric-spatial features. Due to multi-view analysis, the used features are diverse; due to applying fixed convolutional kernels selected from the important regions of the image, deep and informative spatial features compatible with the original data are extracted; and due to the proposed discriminant analysis, the discriminative features with the most differences and minimum redundancy are extracted.
4. The classification map of DFC is highly reliable because it utilizes the confidence maps generated from the neighborhood information of diverse views for decision fusion.

### Multi-view (MW) polarimetric-spatial features

To extract more diverse and richer polarimetric-spatial features, a multi-view classification approach is used in this work. Each pixel of a PolSAR image is generally expressed in the form of a symmetric coherency matrix ${\varvec{T}}$ as follows:

1 $${\varvec{T}}=\left[\begin{array}{ccc}{T}_{11}& {T}_{12}& {T}_{13}\\ {T}_{21}& {T}_{22}& {T}_{23}\\ {T}_{31}& {T}_{32}& {T}_{33}\end{array}\right]$$

where diagonal elements are real numbers and off-diagonal elements are complex ones. So, there are three different types of views in a coherency matrix: view1 = {${T}_{11},{T}_{22},{T}_{33}\}$, which is composed of diagonal elements, view2 = {$Re\left({T}_{12}\right),Re\left({T}_{13}\right),Re\left({T}_{23}\right)\}$, which is composed of real parts of non-diagonal elements and view3 = {$Im\left({T}_{12}\right),Im\left({T}_{13}\right),Im\left({T}_{23}\right)\}$, which is composed of imaginary parts of non-diagonal elements where $Re\left(\cdot \right)$/$Im\left(\cdot \right)$ indicates the real/imaginary part of $\left(\cdot \right)$. The obvious differences among these three views can be effective in increasing the classification accuracy. The classification process is done individually on each view. Finally, the classification results are fused according to the proposed decision fusion rule to result in the final classification map.

The use of spatial information is very important in PolSAR image classification. So, some contextual features are extracted from each view and concatenated to it to form a polarimetric-spatial cube for that view. Because of simple, fast and efficient implementation of morphological filters in extraction of structural and contextual features^32^, the morphological profile (MP) of each view is generated and stacked on it. Therefore, the PolSAR data can be expressed as three views, ${v}_{i}=\left\{{Pol}_{i},{MP}_{i}\right\};i=\mathrm{1,2},3$ where ${Pol}_{i}$ is the polarimetric features of $i$ th view and ${MP}_{i}$ denotes its associated MP.

### Selection of important regions (IRs) as convolutional filters

To extract deeper polarimetric-spatial features containing more robust and informative characteristics, $K$ spatial filters with the size of $W\times W$ are convolved with each view, i.e., ${v}_{i};i=\mathrm{1,2},3$. In contrast to CNN based methods, which need training samples to learn the convolutional filters, the pre-determined convolutional kernels without requirement to learning are used here. To preserve the structure of image and extract features in accordance with the original PolSAR data, the pre-determined convolutional filters are selected from the PolSAR data. A $W\times W$ window around each filter’s center is considered as a convolutional filter. Instead of random selection of $K$ filters’ centers, $K$ pixels of the PolSAR image, which are the key points of data and are in center of the important regions (IRs) are selected. Each view contains ${n}_{v}=3+{n}_{m}$ features where $3$ is the number of polarimetric channels and ${n}_{m}$ is the size of MP in that view. In $i$ th view, only the original polarimetric part containing 3 features, i.e., ${Pol}_{i}=\left\{{P}_{i1},{P}_{i2},{P}_{i3}\right\}$, is used to find the key points. To extract IRs centered in key points, each channel of ${Pol}_{i}$ is smoothed with different scale factors as follows:

2 $${I}_{ij}=\left(G\left(x,y,{\sigma }_{2}\right)-G\left(x,y,{\sigma }_{1}\right)\right)*{P}_{ij} ;i,j=\mathrm{1,2},3$$

where ${I}_{ij}$ is difference of Gaussian of ${P}_{ij}$, $G\left(x,y,\sigma \right)=\frac{1}{2\pi {\sigma }^{2}}{e}^{-\frac{\left({x}^{2}+{y}^{2}\right)}{2{\sigma }^{2}}}$, ${\sigma }_{2}=k{\sigma }_{1}$, $k$ is a constant factor, and ${\sigma }_{1}$ and ${\sigma }_{2}$ are two nearby scales. ${I}_{ij}$ is the difference of smoothed images. Those pixels of ${I}_{ij}$ that have the largest values are the key points indicating obvious features and located in centers of IRs. However, selection of IRs should be done with considering all channels of each view. To this end, each ${I}_{ij}$ is binarized as ${J}_{ij}$ where bright regions of ${I}_{ij}$ are labeled as 1 in ${J}_{ij}$ and others are labeled as 0. Then, ${J}_{ij};j=\mathrm{1,2},3$ regions are fused through the OR logic function to form the index image as ${E}_{i}=\underset{j=\mathrm{1,2},3}{\mathrm{OR}}\left\{{J}_{ij}\right\}$ where $OR$ is the OR logic operator. ${E}_{i}$, as an index image of ${v}_{i}$, is used to fuse ${I}_{ij};j=\mathrm{1,2},3$. The fused image in $i$ th view containing the IRs is computed by:

3 $${IR}_{i}\left(x,y\right)=\left\{\begin{array}{l}\underset{j=\mathrm{1,2},3}{\mathrm{max}}{I}_{ij} ; \quad {E}_{i}\left(x,y\right)=1 \\ \underset{j=\mathrm{1,2},3}{\mathrm{min}}{I}_{ij} ; \quad {E}_{i}\left(x,y\right)=0\end{array}\right.$$

where max/min is the maximum/minimum operator. The convolutional filters in view $i$ are selected from the IR image, i.e., ${IR}_{i}$. The pixels of ${IR}_{i}$ image are sorted in a descending order according to their gray levels. $K$ largest pixels are selected as $K$ key points and considered as centers of $K$ pre-determined convolutional kernels.

Two layers of convolutional filters are applied to each view, ${v}_{i}=\left\{{Pol}_{i},{MP}_{i}\right\};i=\mathrm{1,2},3$. To this end, in the first layer, the principal component analysis (PCA) transform^33^ is applied to ${v}_{i}$ to find the first principal component (PC1) of ${v}_{i}$, indicated by PC1_i_. $K$ filters are applied to PC1_i_ to find $K$ feature maps. In the second layer, the PCA transform is applied to the generated feature cube containing $K$ channels to construct the PC1_i_ in second layer. Then, $K$ convolutional filters are applied to the PC1_i_ to extract $K$ deeper feature maps. The $K$ feature maps generated from the first layer are stacked on the $K$ feature maps generated from the second layer. Then, $2K$ feature maps are concatenated to the initial polarimetric-spatial cube ${v}_{i}=\left\{{Pol}_{i},{MP}_{i}\right\}$ to form a feature cube containing $2K+{n}_{v}$ features. For simplicity in notations, the filtered views, after applying the convolutional kernels, are yet indicated with ${v}_{i}$.

The use of pre-determined convolutional filters have three main advantages: 1. noise reduction, 2. extraction of spatial features accordance with the original PolSAR image, and 3. no requirement to training.

### Two-step discriminant analysis (DA)

According to previous section, ${n}_{b}=2K+{n}_{v}$ polarimetric-spatial features are extracted from each view of the PolSAR data. Due to high dimensionality of the generated polarimetric-spatial cube and also the limited number of training samples, dimensionality reduction is appropriate to do. Feature reduction should be done such a way that not only increases the class discrimination but also avoids overlapping features. In other words, the aim is to generate a reduced polarimetric-spatial cube with minimum redundant information and maximum class separability. To this end, a discriminant analysis is proposed for PolSAR image feature reduction. The proposed method increases the difference between the generated polarimetric-spatial features in the first step (first projection) and maximizes the class discrimination in the second step (second projection) through maximizing the between-class scatters and minimizing the within-class scatters in a nanparametric form.

Let $\mathcal{X}={\left\{{{\varvec{x}}}_{i} : {{\varvec{x}}}_{i}\in {\mathcal{R}}^{{n}_{b}}\right\}}_{i=1}^{{N}_{t}}$ be ${N}_{t}$ training samples belonging to $c$ classes. Class $i$ contains ${n}_{ti}$ training samples where ${N}_{t}=\sum_{i=1}^{c}{n}_{ti}$. The aim is to extract $m$ features from ${n}_{b}$ polarimetric-spatial feature vector corresponding to each pixel ${\varvec{x}}$. With considering the same number of training samples in each class, i.e., ${n}_{ti}={n}_{t};i=\mathrm{1,2},\dots ,c$, corresponding to $j$ th training sample, the training samples matrix of $c$ classes is formed as follows:

4 $${{\varvec{X}}}_{j}=\left[\begin{array}{c}\begin{array}{cc}{x}_{11j}& {x}_{12j}\\ {x}_{21j}& {x}_{22j}\end{array} \begin{array}{cc}\cdots & {x}_{1cj}\\ \cdots & {x}_{2cj}\end{array}\\ \begin{array}{cc}\vdots & \vdots \\ {x}_{{n}_{b}1j}& {x}_{{n}_{b}2j}\end{array} \begin{array}{cc}\cdots & \vdots \\ \cdots & {x}_{{n}_{b}cj}\end{array}\end{array}\right], j=1, 2, \dots , {n}_{t}$$

where ${x}_{ikj}$ $\left(i=1, 2, \dots , {n}_{b}; k=1, 2, \dots , c; j=1, 2, \dots , {n}_{t}\right)$ is $j$ th training sample of class $k$ in $i$ th channel. Corresponding to each channel (each row), the vector ${{\varvec{h}}}_{ij}$ is defined by^34^:

5 $${{\varvec{h}}}_{ij}={\left[\begin{array}{cc}{x}_{i1j}& {x}_{i2j}\end{array} \begin{array}{cc}\cdots & {x}_{icj}\end{array}\right]}^{T} ; i=1, 2,\dots , {n}_{b}, j=1, 2, \dots , {n}_{t}$$

where ${{\varvec{h}}}_{ij}$ contains a representative sample from each class. To extract polarimetric-spatial features with the biggest differences with respect to each other, the between-channel scatter matrix is calculated by:

6 $${{\varvec{S}}}_{\mathrm{c}}=\sum_{j=1}^{{n}_{t}}\sum_{i=1}^{{n}_{b}}\left({{\varvec{h}}}_{ij}-{\overline{{\varvec{h}}} }_{j}\right){\left({{\varvec{h}}}_{ij}-{\overline{{\varvec{h}}} }_{j}\right)}^{T}$$

where ${\overline{{\varvec{h}}} }_{j}=\frac{1}{{n}_{b}}\sum_{i=1}^{{n}_{b}}{{\varvec{h}}}_{ij}$. With maximizing $tr\left({{\varvec{S}}}_{\mathrm{c}}\right)$, the projection matrix ${\varvec{W}}$ is constructed from the eigenvalues of ${{\varvec{S}}}_{\mathrm{c}}$ sorted in a descending order^34^. By applying ${\varvec{W}}$ on the polarimetric-spatial feature space, a new feature space with more differences between polarimetric-spatial channels is generated. According to above transformation, the sample matrix ${{\varvec{X}}}_{j}$ $\left(j=1, 2, \dots , {n}_{t}\right)$ is transformed to the matrix ${\mathcal{R}}_{j}$ as follows:

7 $${\mathcal{R}}_{j}=\left[\begin{array}{c}\begin{array}{cc}{r}_{11j}& {r}_{12j}\\ {r}_{21j}& {r}_{22j}\end{array} \begin{array}{cc}\cdots & {r}_{1cj}\\ \cdots & {r}_{2cj}\end{array}\\ \begin{array}{cc}\vdots & \vdots \\ {r}_{{n}_{b}1j}& {r}_{{n}_{b}2j}\end{array} \begin{array}{cc}\cdots & \vdots \\ \cdots & {r}_{{n}_{b}cj}\end{array}\end{array}\right], j=1, 2, \dots , {n}_{t}$$

To maximize the differences between different classes, a representative vector is defined corresponding to each class as follows:

8 $${{\varvec{R}}}_{kj}={\left[\begin{array}{cc}{r}_{1kj}& {r}_{2kj}\end{array} \begin{array}{cc}\cdots & {r}_{{n}_{b}kj}\end{array}\right]}^{T}$$

Then, the between-class scatter matrix (${{\varvec{S}}}_{\mathrm{b}})$ and within-class scatter matrix ${({\varvec{S}}}_{\mathrm{w}})$ are calculated by:

9 $${{\varvec{S}}}_{\mathrm{b}}=\sum_{j=1}^{{n}_{t}}\sum_{k=1}^{c}\left({{\varvec{R}}}_{kj}-\overline{{\varvec{R}} }\right){\left({{\varvec{R}}}_{kj}-\overline{{\varvec{R}} }\right)}^{T}$$

10 $${{\varvec{S}}}_{\mathrm{w}}=\sum_{k=1}^{c}\sum_{j=1}^{{n}_{t}}\sum_{i=1}^{{n}_{t}}\left({{\varvec{R}}}_{ki}-{{\varvec{R}}}_{kj}\right){\left({{\varvec{R}}}_{ki}-{{\varvec{R}}}_{kj}\right)}^{T}$$

where $\overline{{\varvec{R}} }=\frac{1}{c\times {n}_{t}}\sum_{j=1}^{{n}_{t}}\sum_{k=1}^{c}{{\varvec{R}}}_{kj}$. To deal with the singularity of matrix ${{\varvec{S}}}_{\mathrm{w}}$, it is regularized as ${{\varvec{S}}}_{\mathrm{w}}=0.5{{\varvec{S}}}_{\mathrm{w}}+0.5diag({{\varvec{S}}}_{\mathrm{w}})$. Then, the Fisher criterion, $\mathrm{max}tr\left({{{\varvec{S}}}_{\mathrm{w}}}^{-1}{{\varvec{S}}}_{\mathrm{b}}\right)$, is used to obtain the projection matrix for dimensionality reduction. To extract $m$ features, $m$ eigenvectors of ${{{\varvec{S}}}_{\mathrm{w}}}^{-1}{{\varvec{S}}}_{\mathrm{b}}$ associated with $m$ largest eigenvalues of ${{{\varvec{S}}}_{\mathrm{w}}}^{-1}{{\varvec{S}}}_{\mathrm{b}}$ compose the projection matrix. The proposed feature reduction method has some main advantages: 1-it extracts polarimetric-spatial features with maximum differences with respect to each other, i.e., with minimum redundant information, 2- it maximizes the class discrimination, 3- it can extract any arbitrary number of features and 4- it has good efficiency in small sample size situations thanks to nanparametric form of scatter matrices and the regularization technique.

### Decision fusion with high confidence (HC)

According to the proposed discriminant analysis method in previous section, $m$ features are extracted from each view of the PolSAR data. The $m$ extracted features of view $i$ $\left({v}_{i}\right)$ are given to a SVM classifier to obtain $i$ th $(i=\mathrm{1,2},3)$ classification map. To increase reliability of classification, a confidence map is constructed from each classification map. To this end, a window with length of $L=2a+1$ where $a$ is an integer $a\ge 1$ is considered around each pixel of the classification map. For a central pixel ${{\varvec{x}}}_{c}$, the confidence $g\left({{\varvec{x}}}_{c}\right)$ is defined as $g\left({{\varvec{x}}}_{c}\right)=\rho \left({{\varvec{x}}}_{c}\right)/\left({L}^{2}-1\right)$ where $\rho \left({{\varvec{x}}}_{c}\right)$ is the number of neighboring pixels that have the same label as ${{\varvec{x}}}_{c}$ and ${L}^{2}-1$ is the number of neighbors. Associated with each classification map, a confidence map is generated. For each pixel of image located in position $\left(x,y\right)$, the final label is determined by:

11 $$l\left(x,y\right)={\left(class\_map\right)}_{{i}^{*}}(x,y)$$

where

12 $${i}^{*}=\mathrm{arg}\underset{i=\mathrm{1,2},3}{\mathrm{max}}{(conf\_map)}_{i}(x,y)$$

where ${\left(class\_map\right)}_{i}$ is $i$ th classification map, ${(conf\_map)}_{i}$ is the $i$ th confidence map and $l\left(x,y\right)$ the label of pixel in position $\left(x,y\right)$.

## Experiments

Three real PolSAR datasets are used for evaluation of classification methods: Flevoland, SanFrancisco and Oberpfaffenhofen. The Flevoland and SanFrancisco datasets are L-band PolSAR images acquired by AIRSAR containing15 and 4 classes, respectively. Their sizes are also 750 $\times$ 1024 and 900 $\times$ 1024 pixels, respectively. The Oberpfaffenhofen PolSAR image acquired by electronically steered array radar (ESAR) L-band over Oberpfaffenhofen in Germany has 1297 $\times$ 935 pixels and four classes. A laptop with 2.8 GHz processor, Inter Core i7 CPU, and 16 GB RAM is used for doing experiments. All programs are run by MATLAB 2018b.

### Parameter settings

To provide the initial polarimetric-spatial features in each view, a MP with size of 35 is generated and stacked on the polarimetric features to provide ${v}_{i}=\left\{{Pol}_{i},{MP}_{i}\right\};i=\mathrm{1,2},3$. Each MP consists of 17 channels generated by applying closing operators by reconstruction, 17 associated opening channels and the first principal component of that view. The parameters of the proposed DFC method are set as follows. For the difference of Gaussian function, we set ${\sigma }_{1}=3$, $k=1.5$ $\left({\sigma }_{2}=k{\sigma }_{1}\right)$ and the size of the Gaussian filter equal to $3\times 3$ in all datasets. For extraction of deep features using pre-determined convolutional filters, two layers of convolutional filters with $K=8$ filters in each layer are used. The size of convolutional filters $\left(W\times W\right)$ is considered as a free parameter which is determined for each dataset through doing experiments. The number of features $\left(m\right)$ extracted by using the proposed discriminant analysis and the size of neighboring windows $\left(L\times L\right)$ for generation of confidence maps are also considered as free parameters, which are set for each dataset through doing experiments.

**Figure 1:**
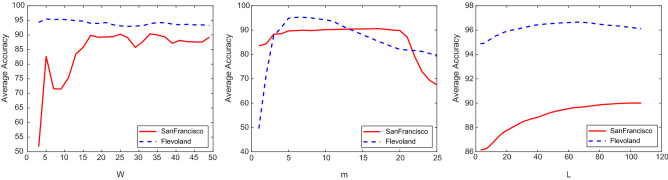
Average classification accuracy versus the free parameters ($W$, $m$ and $L$) of DFC.

Figure 1 shows the average classification accuracy versus the free parameters ($W$, $m$ and $L$) for Flevoland and SanFrancisco datasets. The results are obtained by using 10 training sampler per class. The following conclusions can be found from this figure:

1. Involving spatial information, by applying convolutional kernels selected from the important regions of PolSAR image, improves the classification accuracy. With increasing the size of convolutional filter ($W$), the classification accuracy is improved to a point. But, after that, with increasing the size of filter, it is possible that pixels from the heterogeneous regions or class boundaries are included, which may degrade the classification result. The Flevoland dataset has less sensitivity to the $W$ variations with respect to the SanFrancisco data. For Flevoland dataset, $W=5$ and for SanFrancisco dataset, $W=33$ is selected.
2. With increasing the number of extracted features, the discriminant ability of data is improved and thus, the classification accuracy is increased. But, from a point to next, with increasing the number of extracted features, redundant features are included that may decrease the classification accuracy. $m=7$ and $m=17$ provide the best results for Flevoland and SanFrancisco datasets, respectively.
3. With increasing the size of neighborhood window $\left(L\right)$, with involving more spatial information, the classification accuracy is increased. But, with more increasing of $L$, because of involving pixels belonging to the different classes with respect to the central pixel, the classification result is degraded. The best parameter of $L$ for Flevoland/SanFrancisco data is obtained as $L=63$/$L=105$.

The free parameters in the Oberpfaffenhofen image are set as the same as the Flevoland dataset.

### Assessment of different cases

The proposed DFC method is composed of four main parts: multi-view (MW) analysis, important regions (IRs) selection for convolutional filtering, discriminant analysis (DA) and high confidence (HC) decision fusion. To assess impact of each part, efficiency of the proposed DFC method is compared with the following methods:

- DFC-NMW (DFC without MW): total features composed of diagonal parts, real parts and imaginary parts of non-diagonal elements of the coherency matrix are used together in a single view.
- DFC-NDA (DFC without DA): no discriminant analysis is done in the DFC method.
- DFC-NIR (DFC without IR): instead of selection of important regions, random regions are selected and used as convolutional kernels for spatial filtering.
- DFC-NHC (DFC without HC): instead of using confidence map for decision fusion, the majority voting rule is used for decision fusion.

The classification results for Flevoland dataset achieved by using 100 training samples per class are reported in Table 1. Average accuracy (AA), overall accuracy (OA), kappa coefficient (K) and running time (seconds) are represented. As seen, the lowest classification accuracy is obtained by DFC-NDA, which means that the proposed discriminant analysis has significant impact on the classification result. After DFC-NDA, DFC-NHC results in the lowest classification accuracy, which shows the significant role of the proposed decision fusion. After DA and HC, MW and IR rank third and fourth, respectively, from the significant role point of view. With comparison the running time of different methods, it can be seen that DA is very effective to decrease the running time.

**Table 1:** Classification results for Flevoland dataset achieved by using 100 training samples per class.

Name of class	DFC	DFC-NMW	DFC-NDA	DFC-NIR	DFC-NHC
Stembeans	99.56	99.74	83.27	99.46	99.39
Peas	98.83	99.32	96.21	98.01	98.32
Forest	99.92	99.17	99.31	99.77	98.31
Lucerne	94.68	99.21	91.04	94.61	98.13
Wheat	99.15	97.33	77.70	99.13	97.91
Beet	98.69	95.56	63.05	98.85	96.34
Potatoes	96.55	94.45	95.63	95.12	94.14
Bare soil	100.00	100.00	100.00	100.00	100.00
Grass	99.22	97.08	80.67	99.25	96.70
Rapeseed	98.16	95.34	99.65	97.17	90.72
Barely	99.80	99.58	97.69	100.00	99.23
Wheat 2	99.01	97.68	33.64	97.17	93.02
Wheat 3	99.32	98.39	52.33	99.13	96.40
Water	99.98	99.41	99.38	99.99	97.90
Buildings	99.79	100.00	95.59	100.00	100.00
**AA**	**98.84**	**98.15**	**84.34**	**98.51**	**97.10**
**OA**	**98.72**	**97.77**	**81.40**	**98.31**	**96.52**
**K**	**98.61**	**97.57**	**79.75**	**98.15**	**96.20**
**Time**	**152.45**	**69.51**	**429.06**	**150.75**	**89.16**

To show the statistical significant of differences in the classification results, the McNemars test results are represented in Table 2. According to this table, preference of DFC with respect to other methods is statistically significant with a large difference.

**Table 2:** McNemars test results for Flevoland dataset achieved by using 100 training samples per class.

	DFC	DFC-NMW	DFC-NDA	DFC-NIR	DFC-NHC
DFC	0	26.26	161.98	19.48	52.04
DFC-NMW	− 26.26	0	150.56	− 14.28	28.04
DFC-NDA	− 161.98	− 150.56	0	− 158.30	− 139.35
DFC-NIR	− 19.48	14.28	158.30	0	41.48
DFC-NHC	− 52.04	− 28.04	139.35	− 41.48	0

**Figure 2:**
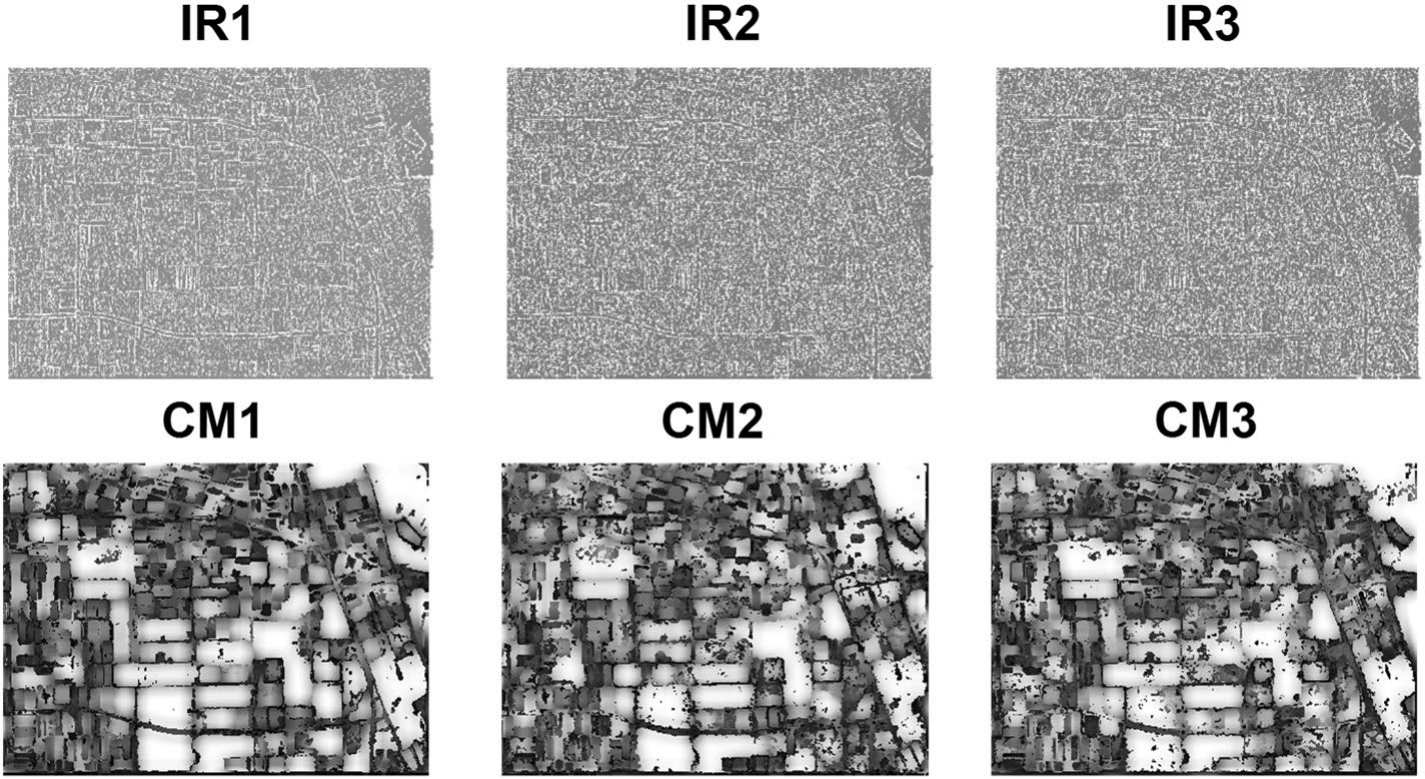
Important regions (IRs) and confidence maps (CMs) for three views of Flevoland dataset.

**Figure 3:**
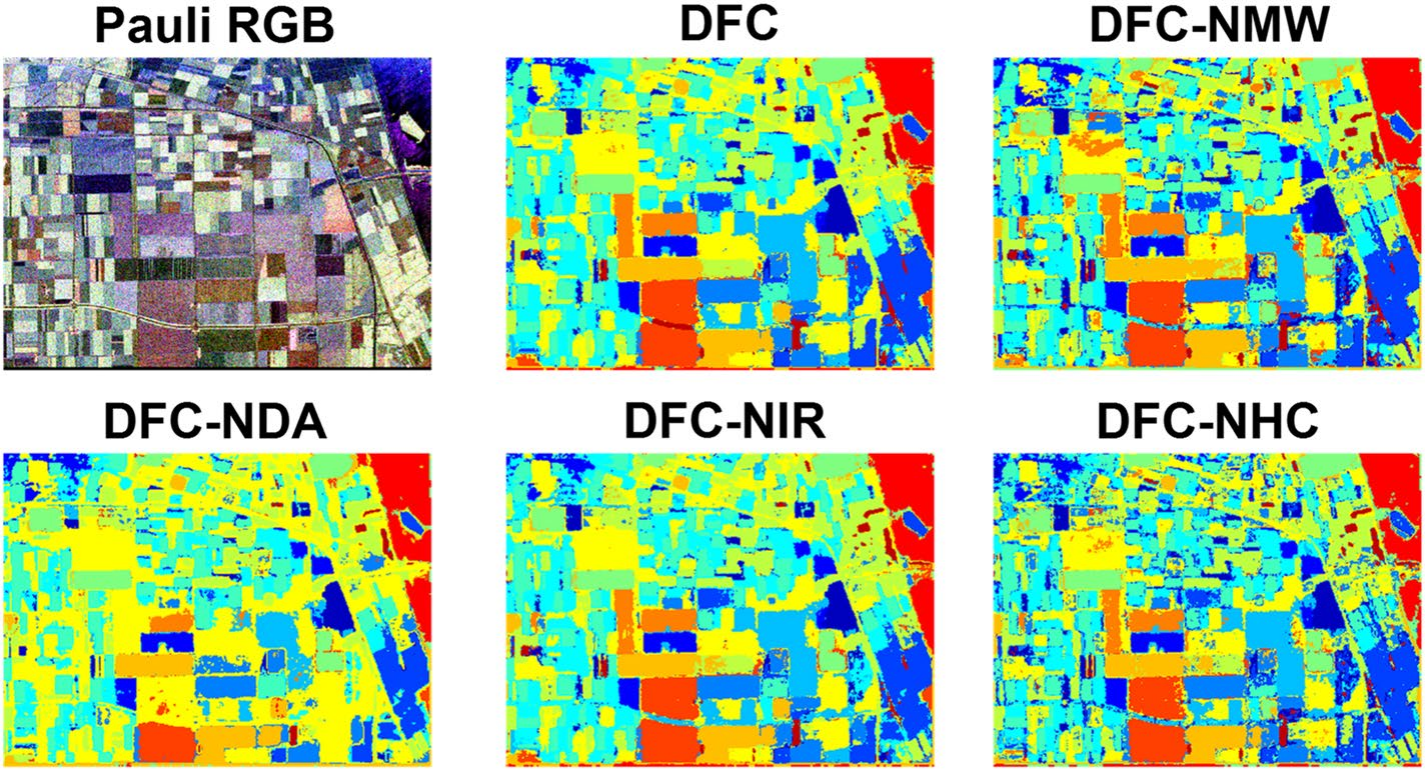
Pauli RGB and the achieved classification maps for Flevoland dataset.

Figure 2 shows the important regions (IRs) and confidence maps (CMs) for three views of Flevoland dataset. The Pauli RGB and the achieved classification maps are also shown in Fig. 3.

**Figure 4:**
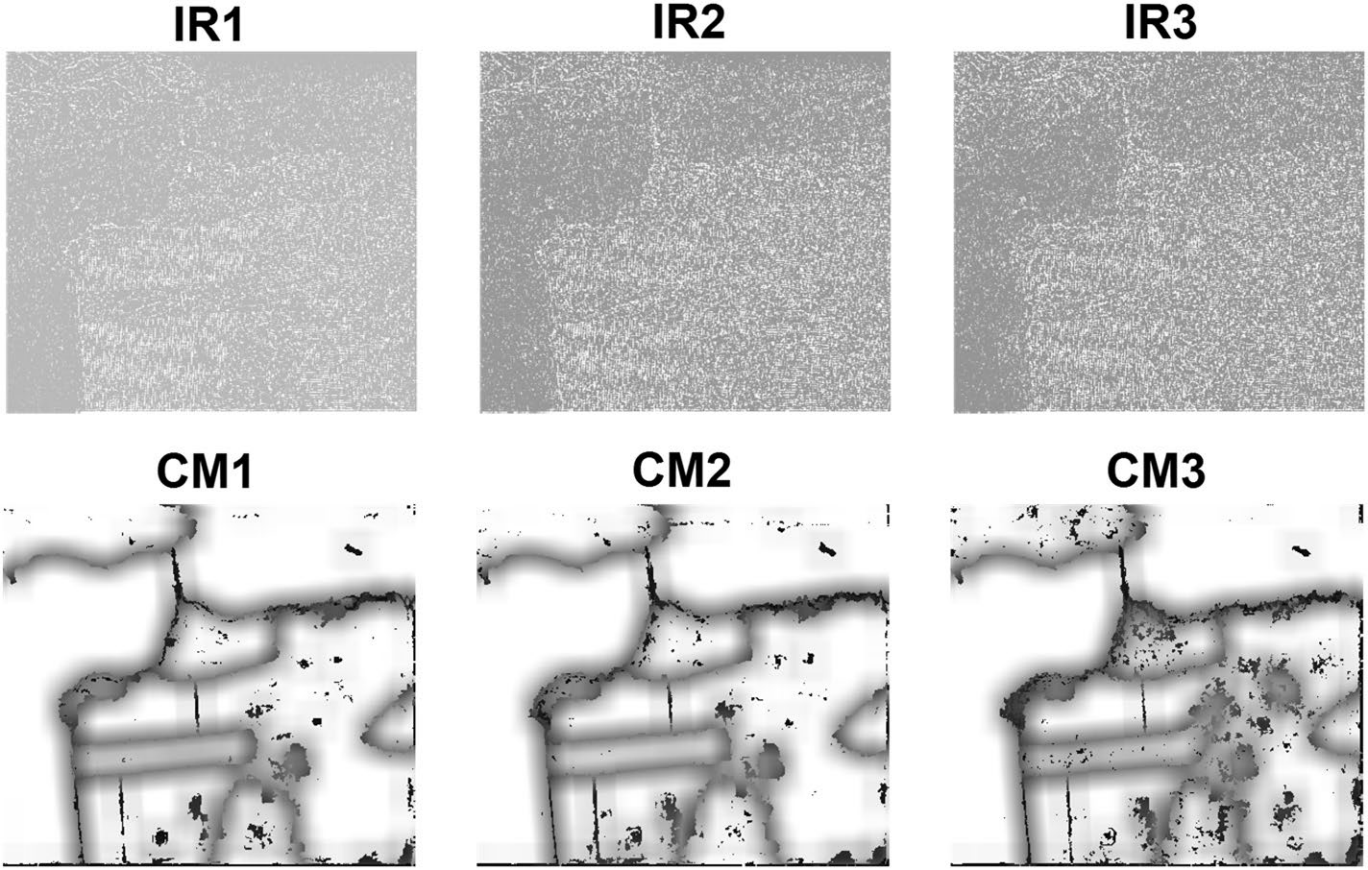
Important regions (IRs) and confidence maps (CMs) for three views of SanFrancisco dataset.

**Figure 5:**
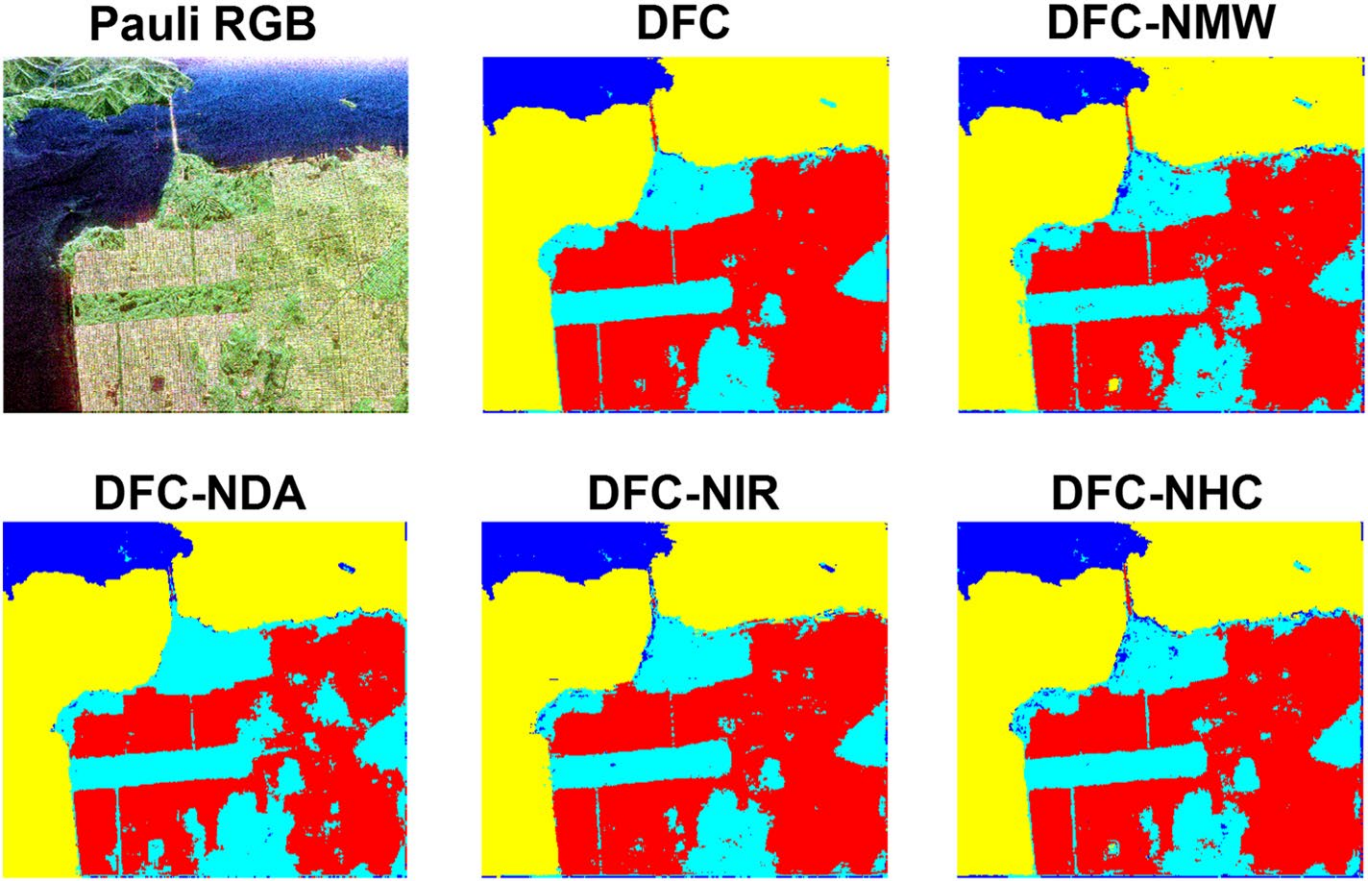
Pauli RGB and the achieved classification maps for SanFrancisco dataset.

The classification results for SanFrancisco dataset by using 750 training samples per class are represented in Table 3 and the McNemars test results are reported in Table 4. According to this table, the important roles for improvement of classification accuracy are under taken by DA, MW, HC and IR, respectively. The McNemars test results show the significant preference of DFC with respect to the competitors from the statistical point of view. Figures 4 and 5 show IRs and CMs and the classification maps, respectively.

**Table 3:** Classification results for SanFrancisco dataset achieved by using 750 training samples per class.

Name of class	DFC	DFC-NMW	DFC-NDA	DFC-NIR	DFC-NHC
Mountain	99.30	98.75	98.06	98.89	99.10
Grass	89.59	89.49	92.08	89.27	88.51
Sea	98.44	97.72	97.97	97.90	98.02
Building	89.33	86.73	78.81	89.06	86.98
**AA**	**94.16**	**93.17**	**91.73**	**93.78**	**93.15**
**OA**	**93.48**	**92.10**	**89.30**	**93.10**	**92.19**
**K**	**90.32**	**88.34**	**84.40**	**89.77**	**88.48**
**Time**	**528.62**	**322.85**	**1090.56**	**555.49**	**332.63**

**Table 4:** McNemars test results for SanFrancisco dataset achieved by using 750 training samples per class.

	DFC	DFC-NMW	DFC-NDA	DFC-NIR	DFC-NHC
DFC	0	69.43	163.12	22.84	75.26
DFC-NMW	− 69.43	0	103.46	− 45.11	− 4.99
DFC-NDA	− 163.12	− 103.46	0	− 140.88	− 117.56
DFC-NIR	− 22.84	45.11	140.88	0	44.30
DFC-NHC	− 75.26	4.99	117.56	− 44.30	0

**Figure 6:**
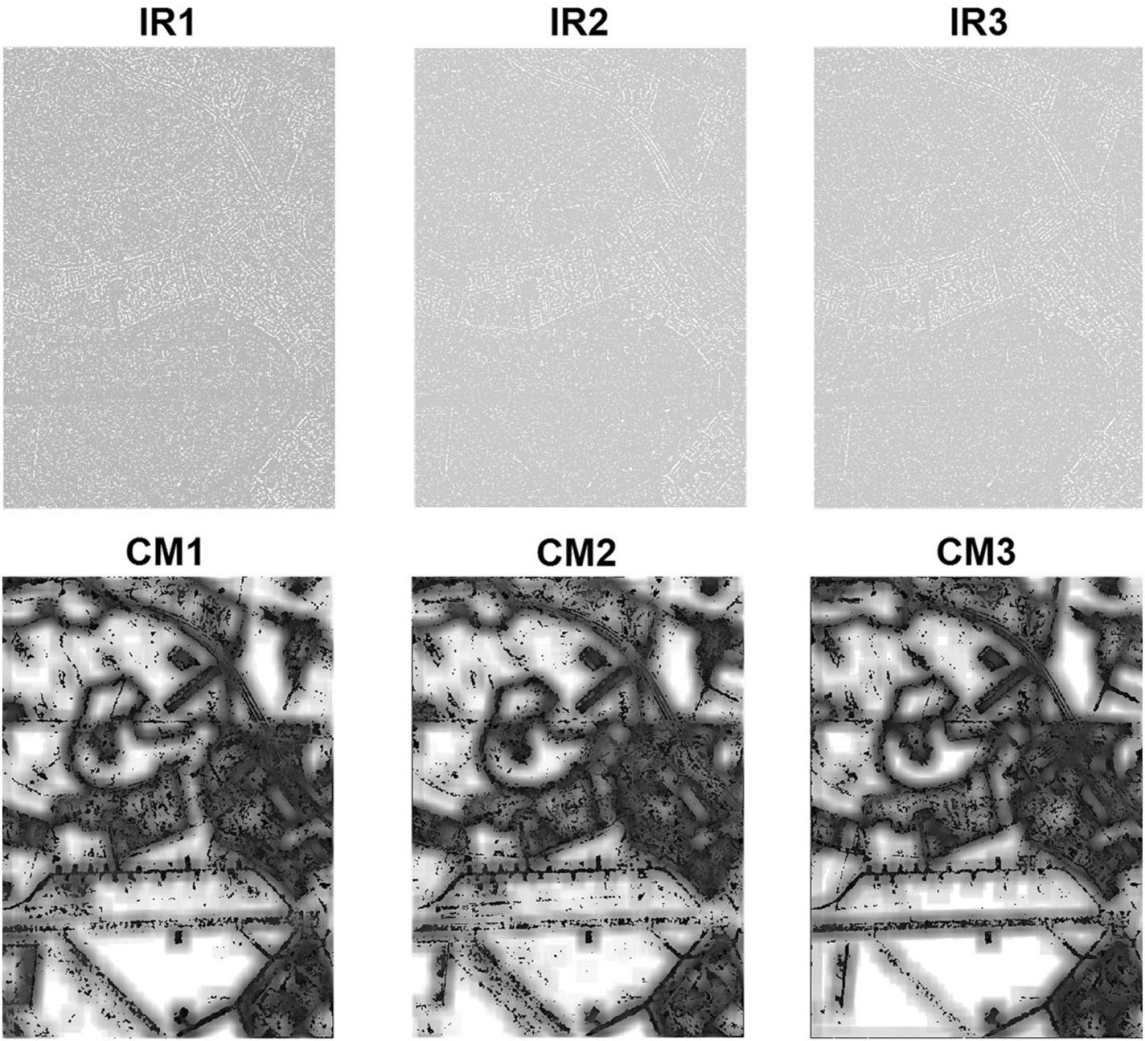
Important regions (IRs) and confidence maps (CMs) for three views of Oberpfaffenhofen dataset.

**Figure 7:**
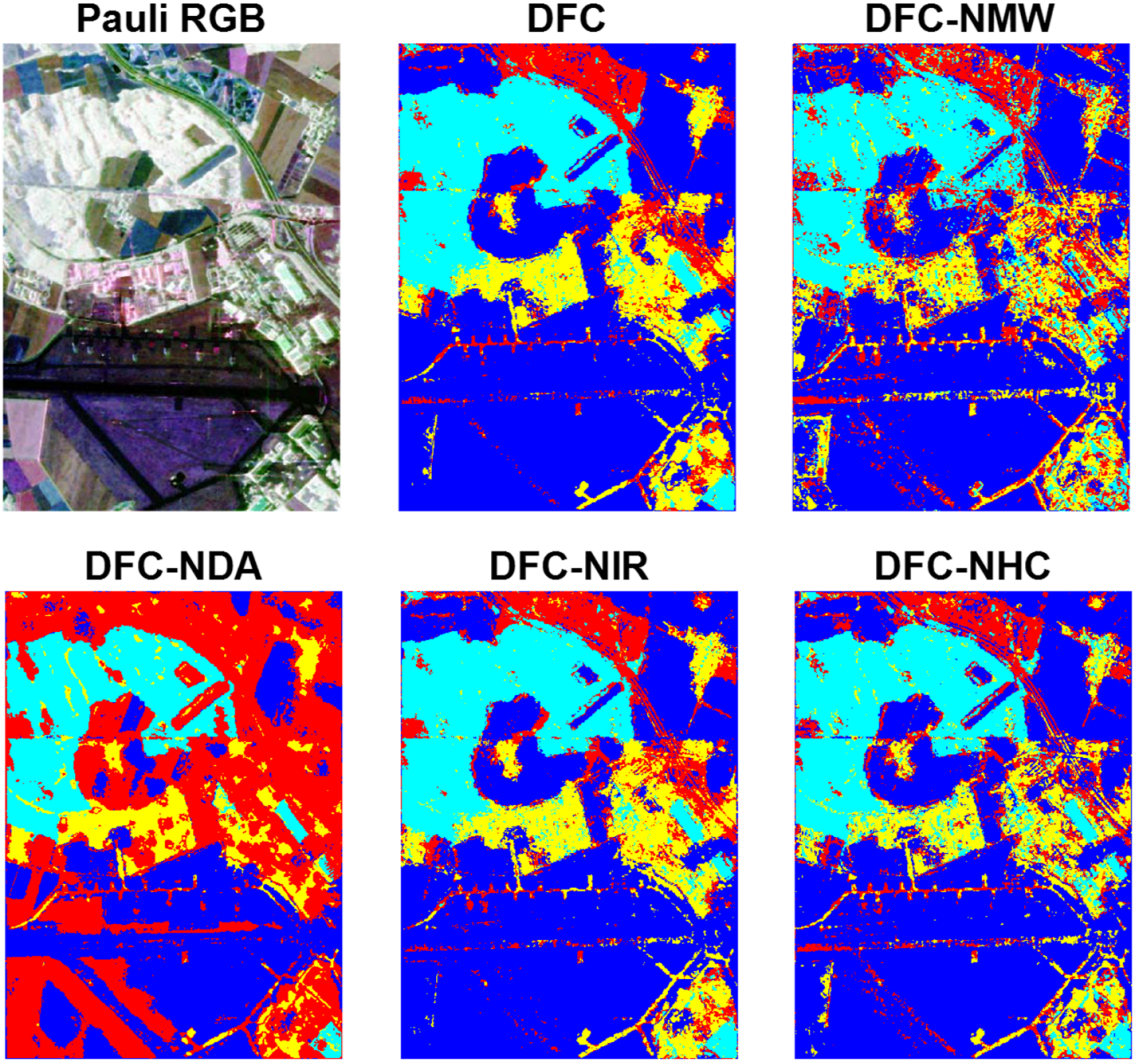
Pauli RGB and the achieved classification maps for Oberpfaffenhofen dataset.

The classification and McNemars test results for Oberpfaffenhofen dataset are reported in Tables 5 and 6, respectively. According to the obtained results, with eliminating the discriminant analysis step for dimensionality reduction, performance of classification is significantly decreased in DFC-NDA. After DA, effects of eliminating the multi-view analysis in DFC-NMW and high confidence decision fusion in DFC-NHC are significant. IRs and CMs and the classification maps for the Oberpfaffenhofen image are shown in Figs. 6 and 7, respectively.

**Table 5:** Classification results for Oberpfaffenhofen dataset achieved by using 100 training samples per class.

Name of class	DFC	DFC-NMW	DFC-NDA	DFC-NIR	DFC-NHC
Open areas	87.64	77.71	51.20	85.00	83.84
Wood land	78.99	72.98	66.52	79.84	75.54
Built-up areas	55.79	43.06	34.02	56.70	44.87
Road	33.85	32.94	68.99	35.77	30.11
**AA**	64.07	56.67	55.18	64.32	58.59
**OA**	72.54	64.27	53.93	71.76	67.69
**K**	57.00	46.05	37.10	56.51	49.74
**Time**	213.58	117.08	369.20	208.06	96.29

**Table 6:** McNemars test results for Oberpfaffenhofen dataset achieved by using 100 training samples per class.

	DFC	DFC-NMW	DFC-NDA	DFC-NIR	DFC-NHC
DFC	0	235.58	357.70	32.56	196.55
DFC-NMW	− 235.58	0	199.84	− 215.85	− 104.44
DFC-NDA	− 357.70	− 199.84	0	− 350.21	− 268.09
DFC-NIR	− 32.56	215.85	350.21	0	147.29
DFC-NHC	− 196.55	104.44	268.09	− 147.29	0

The overall accuracy obtained by using 10 and 100 training samples per class for Flevoland (Fle.) dataset, 10 and 750 training samples per class for SanFrancisco (San.) dataset, and for 10 and 100 training samples per class for Oberpfaffenhofen (Oberp.) dataset are represented in Table 7 for a brief comparison. As we can see, by using limited training samples (the case of 10 samples), the proposed method results in high classification accuracy, which shows superior performance of DFC for dealing with small sample size situations.

**Table 7:** A brief comparison of overall classification accuracy for different cases of DFC method.

Data\method	No of training samples per class	DFC	DFC-NMW	DFC-NDA	DFC-NIR	DFC-NHC
Fle	10	96.40	92.47	69.36	92.81	91.07
	100	98.72	97.77	81.40	98.31	96.52
San	10	90.07	85.04	64.00	82.37	86.21
	750	93.48	92.10	89.30	93.10	92.19
Oberp	10	71.12	64.23	35.23	69.67	67.60
	100	72.54	64.27	53.93	71.76	67.69

### Comparison with other methods

Efficiency of the proposed DFC method is compared with several recently published PolSAR image classification methods. The results are reported in Table 8. The numbers in the table show overall classification accuracy. The numbers in the parentheses are the number of total training samples used in each method indicated by ${N}_{t}$. For the proposed DFC method, the classification results are obtained by using 100 training samples per class for Flevoland dataset, ${N}_{t}=100\times 15=1500$. Also, for SanFrancisco dataset, the results of DFC with 750 training samples per class ${N}_{t}=750\times 4=3000$ are represented. For the method in^10^, result for the best combination of texture and color features (TCF) is reported.

**Table 8:** Comparison of DFC with other classification methods.

Data\method	DFC	TCF^10^	CNN^26^	OA-ELM^17^	CV-3D-CNN^27^	CK-HDRF^11^
Fle	98.72 (1500)	94 (1793)	92.46 (10,817)	93.42 (3000)	93.74 (15,730)	96.75 (1575)
San	93.48 (3000)	92.5 (2500)	90.23 (28,404)	90.44 (3000)	96.86 (9216)	96.56 (9216)

According to Table 8, DFC provides the highest classification accuracy with the lowest number of training samples in Flevoland dataset. In SanFrancisco, DFC outperforms TCF, CNN and OA-ELM. CV-3D-CNN and CK-HDRF with using more training samples result in better classification results compared to others. However, DFC has less sensitivity to the number of training samples because of some reasons: 1. it uses the pre-determined convolutional filters for extraction of deep polarimetric-spatial features, which do not need any training samples or learning process, 2. it uses a nanparametric form of discriminant analysis with regularization technique for feature reduction. In addition, DFC has relatively simple and fast implementation. The running time of DFC by using ${N}_{t}=1500$ is less than 3 min for Flevoland dataset, and by using ${N}_{t}=3000$ is less than 9 min for SanFrancisco dataset.

## Conclusion

A PolSAR image classification method was proposed in this paper that works with a relatively suitable speed and has high classification accuracy especially in small sample size situations. The proposed DFC method applies a multi-view analysis. It appropriately extracts consistent and robust polarimetric-spatial features by applying several pre-determined convolutional kernels. It uses a discriminant analysis through two individual projections. At first, it generates a feature space with minimum overlapping features, and then, it maximizes the class discrimination. Eventually, a high confidence decision fusion is done to provide the final classification map. DFC uses the fixed convolutional kernels for extraction of deep features and also utilizes a nanparametric method for dimensionality reduction. So, DFC has high efficiency in small sample size situations. The convolutional kernels are selected from the important regions of the PolSAR image. The result will be extraction of robust contextual features consistent with the PolSAR inherence. Due to multi-view analysis, DFC utilizes the diverse information of the PolSAR cube. With computing the confidence maps and utilizing them for decision fusion of multi-view classification maps, a high reliable land cover classified map is achieved. The experiments showed that the proposed two-step discriminant analysis feature reduction method has the most important role in classification improvement and in the running time reduction. The important roles of multi-view analysis, selection of important regions and the high confidence based decision fusion were also obvious. By using just 10 training samples per class, DFC provides 96.40%, 90.07% and 71.12% overall classification accuracy in Flevoland, SanFrancisco and Oberpfaffenhofen PolSAR images, respectively. Finally, comparison with other PolSAR classification methods showed the superior performance of DFC in most cases.

## Data Availability

No new data is used in this paper. The datasets used for the experiments are benchmark datasets.
